# Endoscopic closure using a dedicated device following gastric endoscopic submucosal dissection: Multicenter, prospective, observational pilot study

**DOI:** 10.1055/a-2503-1684

**Published:** 2025-01-13

**Authors:** Kazuo Shiotsuki, Kohei Takizawa, Yohei Nose, Yuki Kondo, Hitoshi Homma, Taisuke Inada, Mao Daikaku, Kosuke Maehara, Shin-ichiro Fukuda, Hironori Aoki, Yorinobu Sumida, Hirotada Akiho, Jiro Watari, Kiyokazu Nakajima

**Affiliations:** 1 37060 Department of Gastroenterology, Kitakyushu Municipal Medical Center, Kitakyushu, Japan; 2 91321 Gastroenterology, Kanagawa Cancer Center, Yokohama, Japan; 3 Department of Gastroenterology and Endoscopy, Koyukai Shin-Sapporo Hospital, Sapporo, Japan; 4 Gastroenterological Surgery, Osaka University Graduate School of Medicine, Suita, Japan; 5 Department of Gastroenterological Surgery, Osaka University Graduate School of Medicine, Suita, Japan

**Keywords:** Endoscopy Upper GI Tract, Endoscopic resection (ESD, EMRc, ...), Barrett's and adenocarcinoma, Precancerous conditions & cancerous lesions (displasia and cancer) stomach

## Abstract

**Background and study aims:**

Development of a simple, optimized closure method for mucosal defects left by gastric endoscopic submucosal dissection (ESD) is warranted. Herein, we developed a novel and dedicated closure device called FLEXLOOP and aimed to assess feasibility and safety of the closure using FLEXLOOP following gastric ESD.

**Patients and methods:**

This multicenter, prospective, observational study enrolled patients clinically diagnosed with gastric neoplasms < 30 mm in size. Following gastric ESD, closure of the mucosal defect was performed using a FLEXLOOP with standard clips. The primary outcome was the complete closure rate. The secondary outcomes were procedure time, number of clips, sustained closure rate on second-look endoscopy on postoperative days (PODs) 5 to 7, and rate of post-ESD bleeding.

**Results:**

Overall, 35 patients were included in this study. The median specimen size was 32 mm. The mucosal defect was completely closed in 31 patients (89%; 95% confidence interval, 73%-99%) and incompletely closed in four patients (11%). Median closure time was 11 minutes and median number of clips was 10. Second-look endoscopy performed on PODs 5 to 7 demonstrated sustained, partially sustained, and unsustained closures in seven (20%), 22 (63%), and six patients (17%), respectively. Post-ESD bleeding and complications related to FLEXLOOP were not observed.

**Conclusions:**

Closure using FLEXLOOP is feasible and safe. Our technique using this new device can be an attractive option for more easily closing mucosal defects. However, further clinical research is warranted to confirm that this technique can prevent delayed complications.

## Introduction

Endoscopic submucosal dissection (ESD) is an established specialized technique that enables en bloc resection of neoplasia 1 . With the advancement of technology 2 and expanded indications for treatment 3 4 , ESD for early gastric cancer (EGC) has spread worldwide, and its long-term outcomes are acceptable as a standard treatment instead of gastrectomy 5 .

However, adverse events (AEs), such as post-ESD bleeding or delayed perforation, have yet to be eliminated. In post-ESD bleeding, the risk is 11.45% to 29% in high- to very high-risk cases and should not be ignored as an ESD-related complication 6 , and it is a concern to overcome this problem 7 .

Because the ulcer left by gastric ESD remains open, exposure to gastric acid or bile juice induces AEs. Various techniques and special devices have been proposed to close or protect mucosal defects following gastric ESD to reduce risk of such consequences. However, these methods have not been widely disseminated, mainly owing to technical difficulties and/or cost-effectiveness 8 9 10 .

We also reported the efficacy of endoloop closure for mucosal defects following gastric ESD in high-risk patients 11 , but the procedure was not straightforward.

**Fig. 1:**
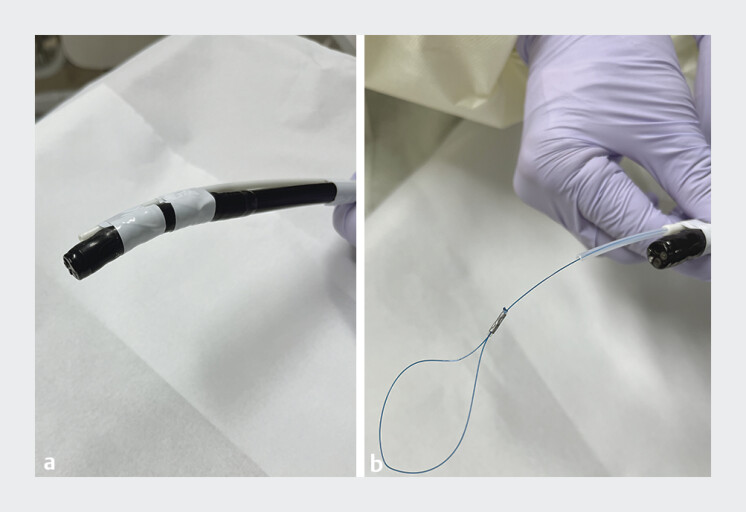
Combination of a single-channel endoscope and FLEXLOOP (Hakko Co., Ltd., Nagano, Japan). The FLEXLOOP comprises a nylon thread and an outer sheath. The nylon thread is joined with silicone rubber and stainless steel.

Endoloop is a device for ligating gastrointestinal polyps, not designed for mucosal closure; therefore, we attempted to develop a dedicated device for closure of mucosal defects. Finally, we developed a novel, simple, and dedicated closure device called FLEXLOOP (Hakko Co., Ltd., Nagano, Japan), consisting of a nylon thread and an outer sheath ( Fig. 1 ) 12 . Clinical feasibility of closure using FLEXLOOP with endoscopic clips has not yet been investigated; thus, this multicenter, prospective, observational pilot study aimed to investigate the feasibility and safety of using FLEXLOOP.

## Patients and methods

### Patients

This study was conducted at Kitakyushu Municipal Medical Center and Koyukai Shin-Sapporo Hospital between November 2022 and August 2023. The current study was approved by each institutional review board in accordance with the Declaration of Helsinki and registered in the Japan Registry of Clinical Trials. All patients provided written informed consent to participate in this study and underwent all the endoscopic procedures.

Inclusion criteria were as follows: 1) a single clinically diagnosed gastric adenoma or EGC < 30 mm in size, which matched the guidelines for ESD and endoscopic mucosal resection for EGC 13 ; 2) age > 20 years; and 3) Eastern Cooperative Oncology Group performance status of 0–2.

If the patients received antithrombotic therapy, we performed ESD by following the guidelines for management of patients receiving antithrombotic therapy 14 .

### Endoscopic submucosal dissection and closure of mucosal defect using FLEXLOOP with endoclips

Complete closure of the mucosal defect following gastric endoscopic submucosal dissection using FLEXLOOP and multiple clips.Video 1

ESD was performed using an ITknife2 (KD-611L; Olympus, Tokyo, Japan) or ORISE ProKnife (M00519361; Boston Scientific Japan, Tokyo, Japan), GIF-290T (Olympus, Tokyo, Japan), a flexible overtube (MD-48518; SB-KWASUMI LABORATORIES, Tokyo, Japan), and a high-frequency generator (VIO3; ERBE, Tubingen, Germany). Radial Jaw Hot Biopsy Forceps (Boston Scientific Japan, Tokyo, Japan) or Hemostat-Y (H-S2518; PENTAX MEDICAL Japan, Tokyo, Japan) was used to perform hemostatic coagulation for intraoperative bleeding and visible vessels on the post-ESD ulcer bed 15 . Although the level of evidence is relatively low, post-ESD coagulation is considered a standard procedure in Japan because of its simplicity and potential for reducing risk of delayed bleeding.

**Fig. 2:**
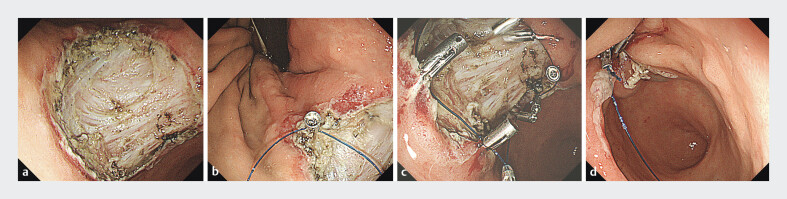
**a** Esophagogastroduodenoscopy reveals a mucosal defect (> 40 mm in diameter) following gastric endoscopic submucosal dissection. **b** The first clip is inserted into the edge of the mucosal defect along with the nylon thread of FLEXLOOP. **c** Multiple clips are circumferentially anchored along the mucosal defect. **d** The mucosal defect is closed by tightening the loop and pushing the outer sheath; subsequently, the mucosal defect is completely closed.

Closure of the mucosal defect using FLEXLOOP with clips (Sure Clip, 11mm; MC Medical, Tokyo, Japan) was performed after gastric ESD. The closure technique involved the following steps. The outer sheath of FLEXLOOP was externally attached on the side of the standard gastrointestinal endoscope. The endoscope was advanced through the overtube, and the loop was deployed and anchored along with the mucosal defect with clips. The defect was circumferentially narrowed with additional several clips, as the loop was tightened by pushing the outer sheath ( Fig. 2 , Video 1 ). After closure, the tail of the loop was cut using endoscopic scissor forceps (FS-410L; Olympus, Tokyo, Japan). All endoscopists were lectured on the closure procedure using FLEXLOOP by watching a video case series. Closure using FLEXLOOP with clips was performed by both experts and nonexperts, with experts defined as board-certified endoscopists.

### Management after ESD

Omeprazole (20 mg/day) was intravenously administered to patients on the day of the ESD procedure and the following day. Laboratory data and physical examinations were performed on postoperative day (POD) 1. A soft food diet and oral potassium-competitive acid blocker (P-CAB) (20 mg/day) or oral proton pump inhibitor (PPI) was started on POD 2 or 3. Second-look endoscopy was performed on PODs 5 to 7 to evaluate closure status. If there were no complications, such as bleeding or perforation, the patients were discharged after POD 8. Oral P-CAB or PPIs were administered for a minimum of 8 weeks, and a third-look endoscopy in the outpatient department was performed 4 or 5 weeks later to assess the ESD site.

### Outcome measurement

The primary outcome was the success rate of complete closure using FLEXLOOP with endoclips. Completeness of closure was divided into three categories: the mucosal defect was completely closed (complete), partially closed (incomplete), or not closed (failure). Complete closure was defined as no ulcer bed visible on endoscopic findings after closure, incomplete closure was defined as slight visibility of the ulcer bed, and failure was defined as closure that could not be performed, and closure was assessed by two endoscopists. Secondary outcomes were procedure time, number of FLEXLOOP, number of clips used, success rate of complete closure related to location or circumference, rate of sustained closure on second-look endoscopy PODs 5 to 7, rate of sustained closure at second-look endoscopy PODs 5 to 7 related to location or circumference, post-ESD bleeding rate, state of closure site approximately 4 or 5 weeks after discharge, and AE -related closure using FLEXLOOP. Closure time was defined as time from opening the loop in the stomach to cutting the loop using the scissor forceps. Sustained closure at second-look endoscopy on PODs 5 to 7 was defined as sustained when the ulcer bed was not visible, partially sustained when the ulcer bed was partially visible, and unsustained when the ulcer bed was fully visible. Post-ESD bleeding was defined as symptoms, such as melena, hematemesis, or decreased hemoglobin level (≥ 2.0 g/dL) that required emergency endoscopy.

### Sample size calculation

Previously, Choi et al. reported that the complete closure rate using only clips was 62% following gastric ESD 16 . Our study group hypothesized that the complete closure rate using FLEXLOOP with clips would be 20% greater than closure using only clips. Based on the parameters α = 0.05 (one-sided level) and power (1-β) = 0.8, a sample size calculation with a one-arm binominal model required 31. Assuming dropout cases, the final target sample size was 35.

## Results

### Patients and ESD procedures

Thirty-five patients were enrolled between November 2022 and August 2023, all of whom underwent ESD and protocol management. There were 27 men and eight women, with a median age of 72 years (range 47–87). Among them, seven patients received antithrombotic therapy and all of them received a single antithrombotic therapy (antiplatelet drug in five patients, anticoagulant drug in two patients). No heparin bridge replacement was performed.

En bloc resection was achieved in all patients, median ESD procedure time was 33 minutes (range 12–107), and no intraoperative or delayed perforation occurred. Median resected specimen and pathological lesion sizes were 32 mm (range 22–56) and 10 mm (range 3–35), respectively. Baseline characteristics and outcomes of ESD are shown in Table 1 and Table 2 , respectively.

**Table:** **Table 1** Baseline characteristics of patients and lesions.

**Characteristics**	**n = 35**
Age, years, median (range)	72 (47–87)
Sex, n (%)	
Men	27 (77)
Women	8 (23)
Comorbidities, n (%)	
Hypertension	14 (40)
Malignancy	10 (29)
Liver disease	3 (9)
Arrhythmia	2 (6)
Ischemic heart disease	2 (6)
Diabetes mellitus	2 (6)
Cerebrovascular disease	1 (3)
Antithrombotic agents, n (%)	
Administered	7 (20)
Not administered	28 (80)
Location, n (%)	
Upper third	4 (11)
Middle third	16 (46)
Lower third	15 (43)
Circumference, n (%)	
Greater curvature	12 (34)
Posterior wall	11 (32)
Lesser curvature	6 (17)
Anterior wall	6 (17)
Gross type, n (%)	
0-IIc	24 (69)
0-IIa	5 (14)
0-IIb	5 (14)
0-IIa + IIc	1 (3)

**Table:** **Table 2** Outcomes of endoscopic submucosal dissection and histology.

	**n = 35**
En bloc resection, n (%)	35 (100)
R0 resection, n (%)	33 (94)
Curative resection, n (%)	33 (94)
Procedure time, median (range), min	33 (12–107)
Size of resected specimen, median (range), mm	32 (22–56)
Size of the tumor, median (range), mm	10 (3–35)
Intraoperative perforation, n (%)	0 (0)
Delayed perforation, n (%)	0 (0)
Histology, n (%)	
Diagnosis	
Adenocarcinoma	35 (100)
Tumor depth	
Mucosa	32 (91)
SM1	1 (3)
SM2	2 (6)
Ulceration	
Absent	35 (100)
Differentiation	
Differentiated	30 (86)
Undifferentiated	5 (14)
Lymphovascular invasion	
Present	1 (3)
Absent	34 (97)

### Outcomes of closure using FLEXLOOP

The mucosal defect was completely closed in 31 patients (89%; 95% confidence interval 73%-99%) and incompletely closed in four patients (11%), and no failures were observed. Median procedure time for closure was 11 minutes (range 8–43), median number of FLEXLOOP was 1 (range 1–2), and median number of clips used was 10 (range 8–17).

Success of complete closure related to location was as follows: upper third, three of four patients (75%); middle third, 14 of 16 patients (88%); and lower third, 14 of 15 patients (93%). Success of complete closure related to circumference was as follows: greater curvature, 10 of 12 patients (83%); posterior wall, 10 of 11 patients (91%); lesser curvature, five of six patients (83%) patients; and anterior wall, six of six patients (100%).

Second-look endoscopy performed on PODs 5 to 7 demonstrated sustained closure in seven patients (20%), partially sustained closure in 22 patients (63%), and unsustained closure in six patients (17%).

Sustained closure on PODs 5 to 7 related to location was as follows: upper third, one of four patients (25%); middle third, three of 16 patients (19%); and lower third, three of 15 patients (20%). Sustained closure on PODs 5 to 7 related to circumference showed the following: greater curvature, three of 12 patients (25%); posterior wall, two of 11 patients (18%); lesser curvature, none of six patients (0%); and anterior wall, two of six patients (33%).

The rate of post-ESD bleeding was 0%. Risk categories for post-ESD bleeding using the BEST-J score prediction model 6 showed that low risk was observed in 27 patients (77%), intermediate risk in five patients (14%), and high risk in three patients (9%).

Two patients were at risk of lymph node metastasis after pathological assessment of ESD specimens; therefore, they underwent additional surgery to prevent distant metastasis. Third-look endoscopy was performed in the remaining 33 patients approximately 4 or 5 weeks after discharge. The mucosal defect developed healing-stage scar formation in 21 patients (64%), the mucosal defect was opened in nine patients (27%), and the mucosal closure remained in three patients (9%). Of the 21 scar formation cases, 19 (90%) had complete closure and two (10%) had incomplete closure; of the nine opened cases, eight (88%) had complete closure and one (12%) had incomplete closure; All three of three remaining cases were complete closures.

AEs related to the procedure using FLEXLOOP with endoclips were not reported. Outcomes of closure using FLEXLOOP with endoclips are shown in Table 3 .

**Table:** **Table 3** Outcomes of closure using FLEXLOOP with endoclips.

	**n = 35**
Completeness of closure using FLEXLOOP and endoclips, n (%)	
Complete	31 (89)
Incomplete	4 (11)
Failure	0 (0)
Procedure time for closure, median (range), min	11 (8–30)
Number of FLEXLOOP, median (range)	1 (1–2)
Number of endoclips, median (range)	10 (8–17)
Adverse events related to closure using FLEXLOOP	0 (0)
Endoscopist degree, n (%)	
Expert	19 (54)
Nonexpert	16 (46)
Sustained closure rate on PODs 5–7	
Sustained	7 (20)
Partially	22 (63)
Unsustained	6 (17)
Complete closure success rate related to location, n (%)	
Upper third	3/4 (75)
Middle third	14/16 (88)
Lower third	14/15 (93)
Complete closure success rate related to circumference, n (%)	
Greater curvature	10/12 (83)
Posterior wall	10/11 (91)
Lesser curvature	5/6 (83)
Anterior wall	6/6 (100)
Sustained closure rate on PODs 5–7 related to location, n (%)	
Upper third	1/4 (25)
Middle third	3/16 (19)
Lower third	3/15 (20)
Sustained closure rate on PODs 5–7 related to circumference, n (%)	
Greater curvature	3/12 (25)
Posterior wall	2/11 (18)
Lesser curvature	0/6 (0)
Anterior wall	2/6 (33)
Post-ESD bleeding, n (%)	0 (0)
Best-J risk stratification, n (%)	
Low risk	27 (77)
Intermediate	5 (14)
High	3 (9)
Closure site at approximately 4 or 5 weeks later, n (%)	
Hearing stage scar formation	21/33 (64)
Closure opened	9/33 (27)
Remained closure	3/33 (9)
ESD, endoscopic submucosal dissection; POD, postoperative day.	

Closure was performed by experts in 19 patients (54%) and by nonexperts in 16 patients (46%). We compared the outcome of closure between experts and nonexperts. Baseline and closure outcome between experts and nonexperts are summarized in Table 4 . No significant differences were observed in location and circumference between experts and nonexperts. Complete closure rates were 84% in experts (16/19) and 94% (15/16) in nonexperts, with no statistically significant difference ( *P* = 0.60). Closure time was longer for experts than for nonexperts ( *P* = 0.04). Tumor size was larger in the expert group than in the nonexpert group, but there was no significant difference between the two groups ( *P* = 0.09).

**Table:** **Table 4** Comparison of closure outcomes using FLEXLOOP with endoscopic clips between expert and nonexpert.

	**Expert n = 19**	**Nonexpert n = 16**	***P* value **
Completeness of closure using FLEXLOOP and endoclips, n (%)			
Complete	16 (84)	15 (94)	0.60
Incomplete	3 (16)	1 (6)	
Failure	0 (0)	0 (0)	
Procedure time for closure, median (range), min	12 (8–30)	9 (8–15)	0.04
Tumor size, median (range), mm	11(4–35)	8 (4–24)	0.09
Location, n (%)			
Upper third	3 (16)	1 (6)	1.00
Middle third	9 (47)	7 (44)	
Lower third	7 (37)	8 (50)	
Circumference, n (%)			
Greater curvature	7 (37)	5 (31)	1.00
Posterior wall	5 (26)	6 (38)	
Lesser curvature	4 (21)	2 (13)	
Anterior wall	3 (16)	3 (18)	
Sustained closure rate on PODs 5–7			
Sustained	1 (5)	6 (38)	0.03
Partially	14 (74)	9 (56)	0.30
Unsustained	4 (21)	1 (6)	0.35
Complete closure success rate related to location, n (%)			
Upper third	2/3 (67)	1/1 (100)	1.00
Middle third	8/9 (88)	6/7(86)	1.00
Lower third	6/7 (86)	8/8 (100)	0.47
Complete closure success rate related to circumference, n (%)			
Greater curvature	6/7 (86)	4/5 (80)	1.00
Posterior wall	4/5 (80)	6/6 (100)	0.46
Lesser curvature	3/4 (75)	2/2 (100)	1.00
Anterior wall	3/3 (100)	3/3 (100)	1.00
Sustained closure rate on PODs 5–7 related to location, n (%)			
Upper third	0/3 (0)	1/1 (100)	0.40
Middle third	0/9 (0)	3/7 (43)	0.06
Lower third	1/7 (14)	2/8 (25)	1.00
Sustained closure rate on PODs 5–7 related to circumference, n (%)			
Greater curvature	1/7 (14)	2/5 (40)	1.00
Posterior wall	0/5 (0)	2/6 (33)	0.45
Lesser curvature	0/4 (0)	0/2 (0)	1.00
Anterior wall	0/3 (0)	2/3 (67)	0.40
POD, postoperative day.			

We investigated risk factors for incomplete closure using FLEXLOOP with endoscopic clips. The details are summarized in Table 5 . Closure time was longer in the incomplete group (14 min) than in the complete group (11 min), and resected specimen size and tumor size were larger in the incomplete group (36 mm and 14 mm) than in the complete group (31 mm and 9 mm), but there was no statistically significant difference between the two groups. There were no statistically significant differences in location or circumference between the incomplete and complete groups.

**Table:** **Table 5** Risk factors for incomplete closure using FLEXLOOP with endoscopic clips.

	**Incomplete n = 4**	**Complete n = 31**	***P* value **
Age, years, median (range)	72 (63–81)	72 (55–84)	1.00
Sex, n (%)			
Men	3 (75)	24 (77)	1.00
Women	1(25)	7 (23)	
Location, n (%)			
Upper third	1 (25)	3 (10)	0.39
Middle third	2 (50)	14 (45)	1.00
Lower third	1 (25)	14 (45)	0.62
Circumference, n (%)			
Greater curvature	2 (50)	10 (32)	0.59
Posterior wall	1 (25)	10 (32)	1.00
Lesser curvature	1 (25)	5 (16)	0.55
Anterior wall	0 (0)	6 (20)	1.00
Endoscopist degree, n (%)			
Expert	3 (75)	16 (52)	0.60
Nonexpert	1 (25)	15 (48)	
Procedure time, median (range), min	14 (9–30)	11(8–21)	0.62
Number of endoclips, median (range)	10 (9–17)	10 (8–17)	0.62
Resected specimen size, median (range), mm	36 (35–56)	31(20–52)	0.60
Tumor size, median (range), mm	14 (3–35)	9(4–30)	0.60

## Discussion

In the present study, we confirmed the feasibility and safety of closure using FLEXLOOP following gastric ESD: The success rate for complete closure was 89%, and no AEs related to closure using FLEXLOOP were reported.

Although closure methods using endoloops have been reported 11 17 , an endoloop is a detachable snare that ligates the stalk of the polyp and is not a closure-dedicated device. Therefore, we developed a closure-dedicated device, FLEXLOOP, whose quality is no less than that of the endoloop and makes it a more simplified device.

Previously, the closure method using endoloop and clips has been reported 11 17 , with closure times of 14 minutes (range 8–47) and 15 minutes (range 4–60), respectively. A previous animal study on closure using FLEXLOOP showed that closure time was shorter than that using an endoloop 12 . Median closure time in this study was 11 minutes (range 8–43), suggesting that closure using FLEXLOOP is also faster than closure using the endoloop. FLEXLOOP consists of an independent outer sheath and nylon thread, which allows flexible adjustment of loop size and position, enabling shorter closure times owing to ease of fixing the loop to the mucosal defect with clips.

The rate of complete closure in the present study was 89%, which was higher than the previous rates of closure using an endoloop of 73% 11 and 86% 17 . Based on these results, we conclude that closure using FLEXLOOP is superior to closure using an endoloop in terms of being a simplified, dedicated closure device, closure time, and rate of complete closure.

As the global population ages, incidence of cardiovascular diseases and arrhythmias has increased, and the number of patients receiving antithrombotic therapy is also increasing 18 . Previous studies have reported an extremely high rate of post-ESD bleeding in patients 19 20 21 22 .

Recently, the BEST-J score has been a predictive model for bleeding risk following gastric ESD 6 , with bleeding risks of 11.4% for high risk and 29.7% for very high risk. Therefore, an effective prophylactic treatment to prevent post-ESD bleeding for high-risk or very-high-risk patients is desired. Although our study included patients at various risks of post-ESD bleeding, we were able to achieve a 0% rate of post-ESD bleeding. In the future, a large prospective study is required to confirm the efficacy of mucosal closure using FLEXLOOP with endoclips in high-risk and very-high-risk patients.

As for the actual number of cases, assuming a post-ESD bleeding rate of 15% in patients with a high or very high BEST-J risk score, we hypothesized that the post-ESD bleeding rate could be reduced to 5% if mucosal closure using FLEXLOOP with endoscopic clips is performed. Based on the parameters α = 0.05 (two-sided level) and power (1-β) = 0.9, a sample size calculation with a one-arm binominal model required 89.

To prevent or reduce risk of AEs, several other closure methods for mucosal defects following gastric ESD have been reported, including closure using the over-the-scope clip (OTSC) system (Ovesco Endoscopy AG, Tubingen, Germany) 8 , closure using OverStich (Apollo Endosurgery Inc., Austin, Texas) 23 , endoscopic hand suturing (EHS) 9 , endoscopic ligation with O-ring closure (E-LOC) 24 , closure using reopenable clip with anchor prongs (Boston Scientific, Marlborough, Massachusetts, United States) 25 , and the clip-over-the-line method (ROLM) 26 . The closure technique using OTSC has a stronger grasping force than the other closure methods but has several problems, such as the possibility of involving other extraluminal organs, high cost, and limited size of the mucosal defect 27 . OverStich is a dedicated suture device produced by Apollo Endosurgery in the United States 23 ; however, in Japan, it is only available at a few facilities and is difficult to use in general hospitals. Moreover, OverStich involves complicated and expensive procedures. EHS is a dedicated suture device that can be domestically used, but it has a time-consuming suturing process (suture time of 49.5 min), involves technical difficulties, and requires expert-level skills 9 . However, in the present study, 46% of the closures were performed by nonexperts and the success rate was 94%. In terms of cost, FLEXLOOP costs USD 46, which is more affordable than other devices, such as EHS, which costs USD 804, or OTSC, which costs USD 534. Hence, closure using FLEXLOOP is simple and cost-effective and does not require special techniques.

E-LOC and ROLM are closure techniques that can be validated in general hospitals using existing endoscopic ligation devices or reopenable clips; however, the procedures seem to be relatively complicated and require a significant amount of procedure time to close within 60 or 30 minutes, respectively.

In terms of closure outcome, rates of complete closure were 91.7% (closure using OTSC), 100% (closure using OverStich), 97% (EHS), 97.9% (E-LOC), 100% (ROLM). The complete closure rate using FLEXLOOP was 89%, which is relatively lower than previous reports, but it is not generally comparable because of differences in endoscopist skills, number of participants, and evaluation methods.

Previous reports on the ulcer healing process have indicated that non-closed ulcers heal in approximately 8 weeks 28 29 . A previous study showed that closure using an endoloop enabled the mucosal defect to heal earlier 30 , and another study examined the healing process of EHS in a porcine model and found that closure of the mucosal defect promoted ulcer healing 31 . Therefore, closure of the mucosal defect potentially promotes ulcer healing. In this study, third-look endoscopy 4 or 5 weeks later revealed that the rate of the mucosal defect developing healing stage was 64%. Thus, our findings are also more supportive of the results of previous studies, which have reported that closure of the mucosal defect suggests that ulcer healing can be promoted.

Although FLEXLOOP is a novel, simple, and dedicated closure device, it has some issues. A previous review article described mucosa-to-mucosa defect closure resulting in submucosal dead space (SDS) due to thickness of the gastric wall, which has been a cause of early phase dehiscence 32 . In our study, the rate of sustained closure on PODs 5 to 7 was 20%, which was potentially due to SDS, but the rate of sustained closure on PODs 10 to 11 in a previous report was 33% 24 , which we believe is comparable with previous results. However, given the low sustained closure rate, we are now planning to improve the closure method using FLEXLOOP to reduce SDS.

Our study has some limitations. First, because this was a pilot study, the sample size was relatively small and there were few lesions in the upper third of the stomach. In general, the likelihood of encountering gastric neoplasia in the upper third is low 7 , and to obtain more lesions in the upper third of the stomach, the total sample size must be larger. Second, because the inclusion criteria in this pilot study were clinically diagnosed gastric neoplasia < 30 mm in size, it is unclear whether mucosal closure using FLEXLOOP with endoclips for lesions > 30 mm in size is feasible. It may be possible to enable closure using a combination of FLEXLOOPs; however, further studies are required. Third, our study did not include lesions extending to the cardia or pyloric ring; therefore, feasibility of closure using FLEXLOOP for these lesions needs to be confirmed in future clinical trials.

## Conclusions

In conclusion, closure of mucosal defects using FLEXLOOP with clips is feasible and safe. Our technique using this new device can be an attractive option for an easier approach to closing mucosal defects. However, further clinical studies are warranted to confirm that this technique can prevent delayed complications.
